# The Budesonide-Hydroxypropyl-β-Cyclodextrin Complex Attenuates ROS Generation, IL-8 Release and Cell Death Induced by Oxidant and Inflammatory Stress. Study on A549 and A-THP-1 Cells

**DOI:** 10.3390/molecules25214882

**Published:** 2020-10-22

**Authors:** Jules César Bayiha, Brigitte Evrard, Didier Cataldo, Pascal De Tullio, Marie-Paule Mingeot-Leclercq

**Affiliations:** 1 Cellular and Molecular Pharmacology Unit, Louvain Drug Research Institute, Université catholique de Louvain, Avenue E. Mounier 73, B1.73.05, 1200 Brussels, Belgium; jules.bayiha@gmail.com; 2 Laboratoire de Technologie Pharmaceutique et Biopharmacie, CIRM, Université de Liège, 4000 Liège, Belgium; B.Evrard@uliege.be; 3 Laboratory of Tumor & Development Biology, GIGA-Cancer, Université de Liège and CHU, 4000 Liège, Belgium; Didier.Cataldo@uliege.be; 4 Laboratoire de Chimie Pharmaceutique, CIRM, Université de Liège, 4000 Liège, Belgium; P.DeTullio@uliege.be

**Keywords:** cyclodextrins, HPβCD, budesonide, inflammation, ROS, Akt, HDAC, cholesterol

## Abstract

Synthetic glucocorticoids such as budesonide (BUD) are potent anti-inflammatory drugs commonly used to treat patients suffering from chronic inflammatory diseases. A previous animal study reported a higher anti-inflammatory activity with a 2-hydroxypropyl-β-cyclodextrin (HPβCD)-based formulation of BUD (BUD:HPβCD). This study investigated, on cellular models (A549 and A-THP-1), the effect of BUD:HPβD in comparison with BUD and HPβCD on the effects induced by oxidative and inflammatory stress as well as the role of cholesterol. We demonstrated the protective effect afforded by BUD:HPβCD against cytotoxicity and ROS generation induced by oxidative and inflammatory stress. The effect observed for BUD:HPβCD was comparable to that observed with HPβCD with no major effect of cholesterol content. We also demonstrated (i) the involvement of the canonical molecular pathway including ROS generation, a decrease in PI3K/Akt activation, and decrease in phosphorylated/unphosphorylated HDAC2 in the effect induced by BUD:HPβCD, (ii) the maintenance of IL-8 decrease with BUD:HPβCD, and (iii) the absence of improvement in glucocorticoid insensitivity with BUD:HPβCD in comparison with BUD, in conditions where HDAC2 was inhibited. Resulting from HPβCD antioxidant and anticytotoxic potential and protective capacity against ROS-induced PI3K/Akt signaling and HDAC2 inhibition, BUD:HPβCD might be more beneficial than BUD alone in a context of concomitant oxidative and inflammatory stress.

## 1. Introduction

Inhaled corticosteroids were first discovered 50 years ago and are used as anti-inflammatory drugs. They are very effective controllers of asthma and largely used in chronic obstructive pulmonary disease (COPD) to prevent exacerbations and improve quality of life in COPD patients [1,2] despite the appearance of corticosteroid insensitivity [3]. Several alternatives to glucocorticoids have been developed in the past few years [4,5,6] but efforts are still essential to address the lack of treatment options in COPD smoking patients for whom a loss of sensitivity to glucocorticoids is observed [7]. The cellular and molecular mechanisms underlying steroid insensitivity in severe asthma and COPD are still not fully understood [7]. Oxidative stress, an increase in phosphoinositide-3-kinase/Akt (PI3K/Akt) signaling leading to the phosphorylation of HDAC2, associated with a loss of HDAC2 activity, could be critical [3,8].

Budesonide (BUD) is one of the most extensively used inhaled glucocorticoids including in the prophylactic management of asthma [9] and smoking-induced COPD [10,11]. However, frequent dosing remains a major concern in the use of budesonide. Moreover, the therapeutic potential of budesonide might be limited by its low solubility at a physiological pH. The development of budesonide formulations that can enhance drug solubility and the dissolution rate in biological fluids will likely achieve higher tissue concentrations and effectiveness.

With the aim to improve the use of inhaled corticoids with sustained release, Dufour et al. [12] evaluated in a mouse model of asthma a new formulation where budesonide was complexed with cyclodextrin (2-hydroxypropyl-β-cycodextrin; HPβCD). In a model of smoking-induced COPD in mammals, Cataldo et al. (Cataldo et al., Patent, 2014) suggested a potential interest in a pharmaceutical preparation resulting from the complexation of budesonide with HPβCD.

Cyclodextrins are typically cone-shaped cyclic oligosaccharides of six (α-CD), seven (β-CD) or eight (γ-CD) glucose units. They possess a hydrophobic cavity allowing them to host hydrophobic molecules. They are widely used as complexing agents for low water-soluble drugs to improve their physicochemical properties including solubility, bioavailability and stability, but they also have many other applications in food, cosmetics, or textiles, for example [13,14,15,16]. β-CD and its derivatives can form a soluble inclusion complex with cholesterol and are often used to extract it from biological material [15]. Among β-CDs, methyl-β-CD (MβCD) is the most effective and the most used method to extract cholesterol but has limited clinical application, unlike HPβCD whose clinical application is broader [17,18,19,20].

The main anti-inflammatory mechanism of glucocorticoids involves the activation of glucocorticoid receptors in the cytosol after glucocorticoid binding, leading to their translocation to the nucleus, where they recruit histone deacetylase 2 (HDAC2) to the activated inflammatory gene complex. HDAC2 then reduces the acetylation of histones and glucocorticoid receptors, allowing chromatin condensation and the trans-repression of inflammatory transcription factors, respectively [21,22]. Through a decrease in activity and expression of HDAC2 in lung airways and alveolar macrophages, corticosteroid treatment is poorly effective for patients suffering from COPD [21,22]. Based on the characterized interaction between cyclodextrins and cholesterol, we hypothesized that this interaction could be involved in the effects of the BUD:HPβCD complex. Cholesterol is largely known for its effect on biophysical membrane properties and cholesterol-enriched domains are linked to membrane signaling [23,24,25,26] including pathways involved in PI3K/Akt signaling and inflammation processes. On giant unilamellar vesicles (GUVs) and lipid monolayers, BUD:HPβCD induced the disruption of cholesterol-enriched raft-like liquid ordered domains—an increase in membrane permeability and fluidity [27]. Except for membrane fluidity, all these effects were enhanced when HPβCD was complexed with budesonide as compared with HPβCD [27]. On cellular models, this could involve signal transduction pathways such as ROS generation, inflammatory cytokines expression and cell death. 

The current study aimed to characterize the effect of the BUD:HPβCD complex in comparison with BUD and HPβCD on the response of human alveolar epithelial cells (A549) or human monocytes (A-THP1) to a mix of hydrogen peroxide and lipopolysaccharide (H_2_O_2_ + LPS) mimicking stressful effects including those from cigarette smoke [28,29,30] or from environmental toxicants. In detail, we pursued four objectives: first, to establish the potential interest of BUD:HPβCD on the cytotoxicity induced by oxidative and inflammatory stressors; second, to investigate the cellular effect of BUD:HPβCD on the signaling pathway involved in corticosteroid effects including ROS generation, PI3K/Akt activation, HDAC2 activity and the release of pro-inflammatory cytokines such as IL-8; third, to question the role of cholesterol in the effect induced by BUD:HPBCD on ROS generation and PI3K/Akt activation, with the two first membranous events leading to inflammation, and fourth, to determine the effect of BUD:HPβCD as compared to BUD in glucocortioid resistance and the role of HDAC2 in mediating the loss of the glucocorticoid anti-inflammatory effect. 

This study is a part of the continuing efforts to develop novel drug delivery systems, as the complex between budesonide and cyclodextrins, with the aim to improve the treatment of patients suffering from smoking-induced COPD.

## 2. Results

### 2.1. BUD:HPβCD Complex and HPβCD Attenuate H_2_O_2_ + LPS-Induced Cytotoxicity

**Figure 1:**
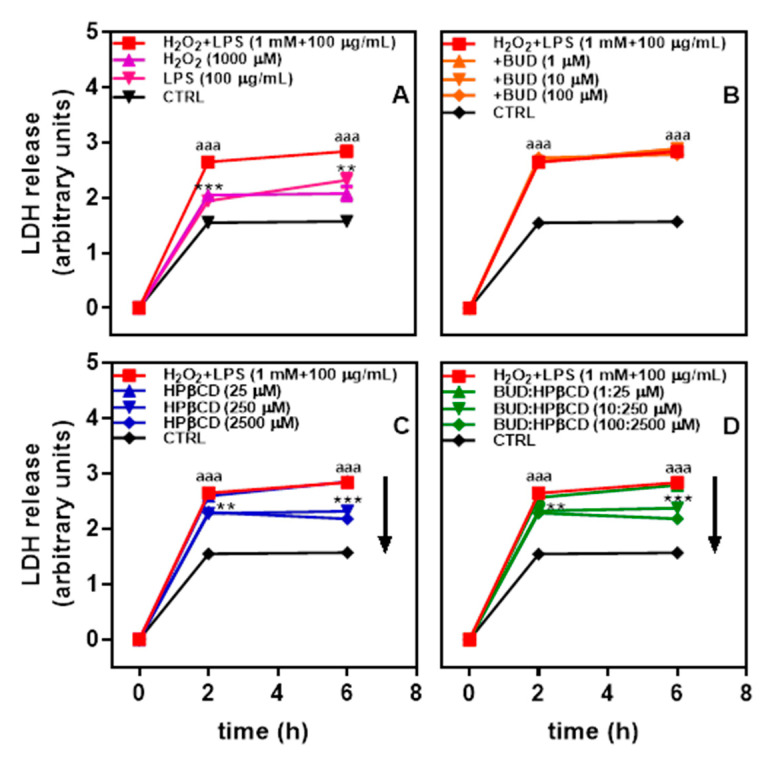
Lactate dehydrogenase (LDH) release after A549 cells incubation with H_2_O_2_, Lipopolysaccharides (LPSs), and H_2_O_2_ + LPS for up to 6 h (**A**) and effect of budesonide (BUD) (**B**), HPβCD (**C**), and BUD:HPβCD complex (**D**) on H_2_O_2_ + LPS-induced cytotoxicity. Each point represents the mean ± SEM of at least 4 independent means of triplicated measures; where not visible, error bars are included in the symbol. The difference was considered significant for a *p*-value < 0.05. (aaa) indicates *p* < 0.001 versus untreated group; (**) and (***) corresponds to *p* < 0.01 and 0.001 versus H_2_O_2_ +LPS-treated group, respectively.

Since alveolar cell death is one feature observed in the lung of patients suffering from smoking-induced COPD [31], the potential effect of BUD:HPβCD on A549 human alveolar epithelial cells submitted to oxidant and inflammatory stressors was investigated. Cells were incubated with H_2_O_2_ + LPS for 2 h. Cytotoxicity, as reflected by lactate dehydrogenase (LDH) release, was observed with a 1.7-fold increase as compared to untreated cells (Figure 1A). The increase in cytotoxicity between 2 and 6 h is low (1.8-fold at 6 h) suggesting the cytotoxicity almost reached its maximum at 2 h. H_2_O_2_ + LPS-induced cytotoxicity seemed to result from the addition of H_2_O_2_ and LPS. 

The effect of the BUD:HPβCD complex on cytotoxicity induced by H_2_O_2_ + LPS was followed. Incubation of A549 cells with the BUD:HPβCD complex together with H_2_O_2_ + LPS induced a decrease in cytotoxicity (Figure 1D). This protective effect appeared to not evolve further after 2 h of incubation. A similar effect was recorded with HPβCD (Figure 1C), whereas BUD showed no effect whatever the dosage (Figure 1B). These results suggest that the BUD:HPβCD complex and HPβCD would have anticytotoxic potential against H_2_O_2_ + LPS-induced cytotoxicity in A549 cells.

To determine if apoptosis is involved in the cell death process for which BUD:HPβCD could protect, apoptosis was monitored by counting condensed/fragmented nuclei using HOECHST dye on H_2_O_2_ + LPS-treated cells. We also determined if the BUD:HPβCD complex as well as BUD, and HPβCD could attenuate apoptosis.

**Figure 2:**
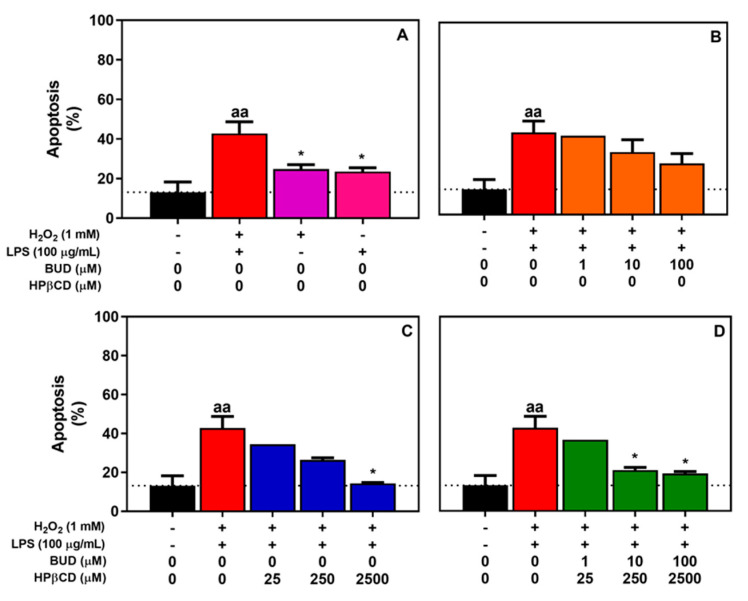
A549 cells apoptosis after treatment with H_2_O_2_, LPS, and H_2_O_2_ + LPS for 2 h (**A**) and effect of BUD (**B**), HPβCD (**C**), and BUD:HPβCD complex (**D**) on H_2_O_2_ + LPS-induced apoptosis. Apoptosis was quantified by counting condensed/fragmented nuclei after HOECHST staining. Each bar represents the mean of 3 ± SEM or 2 independent measures. A one-way ANOVA with Dunett post-test was used to compare the mean of a test group with the mean of the untreated group or H_2_O_2_ +LPS-treated group. The difference was considered significant for a *p*-value < 0.05. (aa) indicates *p* < 0.01 versus untreated group, (*) indicates *p* < 0.05 versus H_2_O_2_ +LPS-treated group.

A549 cells were incubated for 2 h with H_2_O_2_ + LPS with/without BUD:HPβCD in comparison with BUD or HPβCD. H_2_O_2_ + LPS induced significant apoptosis, which appeared to result from the addition of the individual effects of H_2_O_2_ and LPS (Figure 2A). Concomitant incubation with each of the selected compounds induced a concentration-dependent decrease in H_2_O_2_ + LPS-induced apoptosis. These results suggest that the BUD:HPβCD complex (Figure 2D) and HPβCD (Figure 2C), at the highest selected dosages could protect cells against H_2_O_2_ + LPS-induced apoptosis in A549 cells. A non-significant decrease was observed with BUD (Figure 2B).

### 2.2. BUD:HPβCD Complex and HPβCD Protect against H_2_O_2_ + LPS-Induced Oxidative Stress in A549 Cells: Dose and Time-Dependent Effects

Because oxidative stress is critical for numerous pathologies including smoking-induced COPD [32], and with the aim to understand the mechanism of action behind the effects observed with BUD:HPβCD, the potential antioxidant effect of the BUD:HPβCD complex in A549 human alveolar epithelial cells was monitored. A549 cells were incubated for 2 h with a H_2_O_2_ + LPS mix to model a concomitant oxidative and inflammatory environment. ROS generation induced by the BUD:HPβCD complex was monitored in comparison with the effect induced by budesonide or HPβCD.

**Figure 3:**
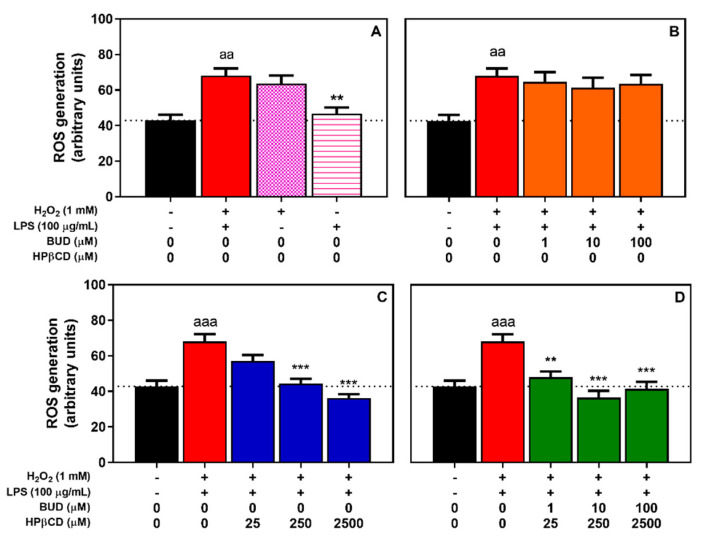
ROS generation in A549 cells after treatment with H_2_O_2_, LPS, or H_2_O_2_ + LPS for 2 h (**A**) and effect of BUD (**B**), HPβCD (**C**), and BUD:HPβCD complex (**D**) on H_2_O_2_ + LPS-induced ROS generation. ROS generation was evaluated by measuring the fluorescence of dichlorofluorescein (DCF). Each bar represents the mean ± SEM of 4 independent means of triplicated measures. A one-way ANOVA with Dunett post-test was used to compare the mean of a test group with the mean of untreated group or H_2_O_2_ + LPS-treated group. The difference was considered significant for a *p*-value < 0.05. (aa), and (aaa), indicate *p* < 0.01, and 0.001 versus untreated group, respectively); (**), and (***), correspond to *p* < 0.01 and 0.001 versus H_2_O_2_ + LPS-treated group, respectively.

In comparison with control cells, we observed a 1.6-fold significant increase in intracellular ROS production when cells were incubated with H_2_O_2_ + LPS (Figure 3A) This effect was similar to the effect induced by treatment with H_2_O_2_ alone (1.5-fold increase), unlike the treatment with LPS alone, which did not show any significant effects, suggesting that H_2_O_2_ + LPS-induced oxidative stress would be mainly driven by H_2_O_2_ in A549 cells.

A concomitant incubation of A549 cells with H_2_O_2_ + LPS and increasing concentrations of the BUD:HPβCD complex (1:25, 10:250, 100:2500 µM; Figure 3D) was associated with a decrease in ROS production as compared with the experimental conditions in which the complex was not present. This suggests a protective effect of the BUD:HPβCD complex against the oxidative stress induced by H_2_O_2_ + LPS. A similar effect was observed with increasing concentrations of HPβCD (25–2500 µM; Figure 3C). In contrast no effect of BUD (1–100 µM; Figure 3B) was observed. 

**Figure 4:**
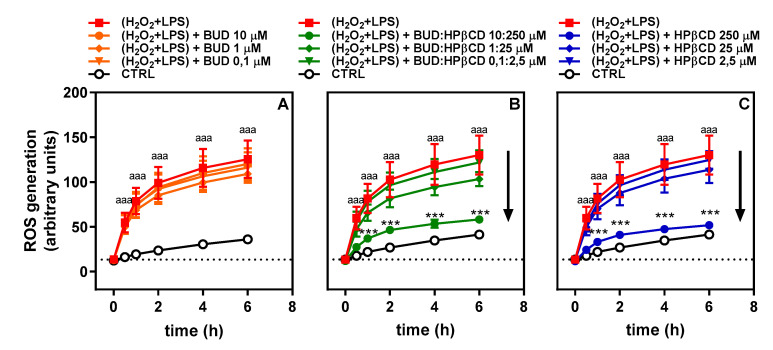
Effect of BUD (**A**), BUD:HPβCD complex (**B**) and HPβCD (**C**) on H_2_O_2_ + LPS-induced ROS generation for 0 to 6h of incubation. ROS generation was evaluated by measuring the fluorescence of dichlorofluorescein (DCF). Each bar represents the mean ± SEM of 3 independent means of triplicated measures. These results and the results illustrated in Figure 3 are independent. When deviations are not visible they are too small to be seen. The difference was considered significant for a *p*-value < 0.05. (aaa) corresponds to *p* < 0.001 versus untreated group; (***) indicates *p* < 0.001 versus H_2_O_2_ + LPS-treated group.

Regarding the effect of time (Figure 4), ROS production in the presence of H_2_O_2_ + LPS was already marked after 30 min compared to untreated cells. This effect was maintained throughout the entire time period investigated (6 h) and seemed to evolve in parallel with untreated cells after 2 h. When the BUD:HPβCD complex was added together with H_2_O_2_ + LPS, a lowering effect on ROS production was observed throughout the entire time period investigated (Figure 4B). The extent of the effect depended upon the dose and was largely similar to the effect observed in the presence of HPβCD (Figure 4C). No significant change was observed after 2 h of incubation with H_2_O_2_ + LPS. Again, during the entire period investigated, no significant effect was observed in the presence of BUD (Figure 4A). Altogether, these results suggest that the BUD:HPβCD complex and HPβCD have a similar antioxidant potential against H_2_O_2_ + LPS in A549 cells.

### 2.3. BUD:HPβCD Complex and HPβCD Attenuate H_2_O_2_ + LPS-Induced Phosphoinositide-3-Kinase/Akt Signaling in A549 Cells

**Figure 5:**
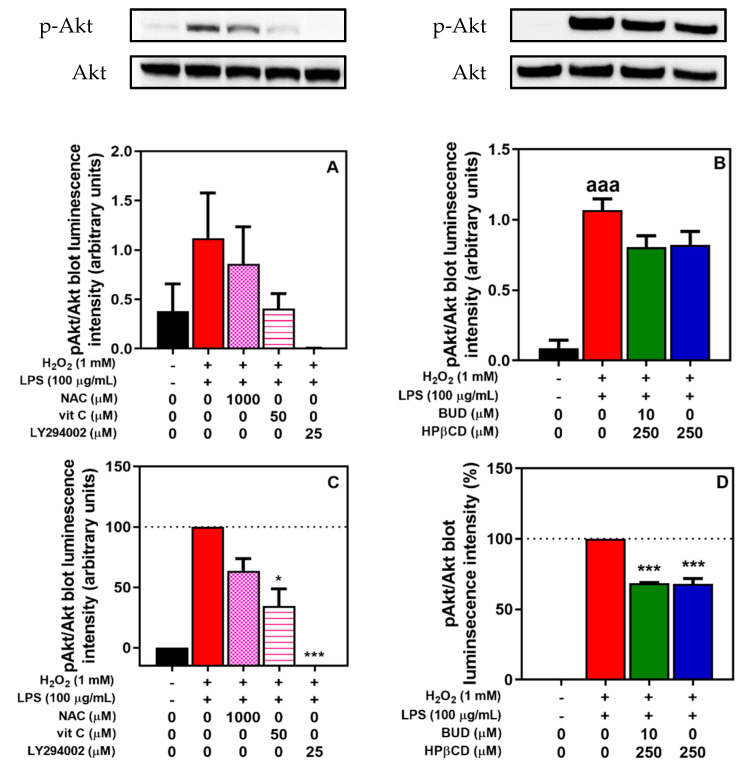
Akt phosphorylation induced by H_2_O_2_ + LPS in A549 cells. Effect of *N*-acetyl-L-cysteine (NAC), vitamin C (VitC) and LY294002 (**A**,**C**) and BUD:HPβCD complex and HPβCD (**B**,**D**) after 2 h of incubation. Data are expressed in absolute values (A/B; with representative blots) or in relative values (in comparison with the pAkt/Akt ration of cells incubated with H_2_O_2_ + LPS; C/D). Akt phosphorylation was quantified after a Western blot by measuring the proportion of phosphorylated-Akt (p-Akt) blot luminescence intensity/total Akt (Akt) blot luminescence intensity. Each bar represents the mean of 3 independent measures. A one-way ANOVA with Dunett post-test was used to compare the mean of each test group with the mean of untreated group or H_2_O_2_ + LPS-treated group. The difference was considered significant for a *p*-value < 0.05. (aaa) correspond to *p* < 0.001 versus untreated group, (*) and (***) indicate *p* < 0.05, and 0.001 versus H_2_O_2_ + LPS-treated group, respectively.

Oxidative stress-induced glucocorticoid insensitivity involves an increase in PI3K/Akt signaling [8,33,34] as reflected by Akt phosphorylation. To validate in in vitro model the relationship between oxidative stress and increase in PI3K/Akt signaling, the phosphorylation of Akt, in the absence or in the presence of antioxidants (N-acetyl-L-cysteine (NAC), vitamin C (Vit C)) and of a PI3K inhibitor (LY294002) (Figure 5A,C) was measured. An incubation of A549 cells with H2O2 + LPS for 2 h increased Akt phosphorylation. Concomitant incubation with NAC or vitamin C or LY294002 was associated with a lower phosphorylation of Akt (of approximately 36% (NAC) and 65% (Vit C)) or a complete suppression of phosphorylation (LY294002) (Figure 5A,C). Regarding the effect of the BUD:HPβCD complex or of HPβCD at a concentration at which a significant antioxidant effect was observed, we demonstrated a decrease in Akt phosphorylation (Figure 5B,D). The decrease was approximately 32% both for the BUD:HPβCD complex and for HPβCD (Figure 5B,D). Thus, the BUD:HPβCD complex and HPβCD inhibited H2O2 + LPS-induced PI3K/Akt signaling increases in a similar way in A549 cells.

### 2.4. Cholesterol Might Limit the Effects of BUD:HPβCD Complex and HPβCD in ROS Generation and PI3K/Akt Signaling Induced by H_2_O_2_ + LPS

To give insight on the molecular mechanisms involved in the protective effect of BUD:HPBCD and HPBCD, the potential role of cholesterol on ROS generation and PI3/Akt phosphorylation induced by H_2_O_2_ + LPS as well as the protective effects of the BUD:HPβCD complex and HPβCD were investigated. The rationale was derived from the ability of cyclodextrins to interact with cholesterol [35], the effects of BUD:HPβCD and HPβCD on the biophysical membrane properties of cholesterol-enriched domains [27], the importance of lipid-ordered domains enriched in cholesterol in membrane called rafts for ROS generation [36,37] and PI3K/Akt signaling [23,26].

#### 2.4.1. Cholesterol Content Might Influence the Effects of the BUD:HPβCD Complex and HPβCD in ROS Generation Induced by H_2_O_2_ + LPS

**Figure 6:**
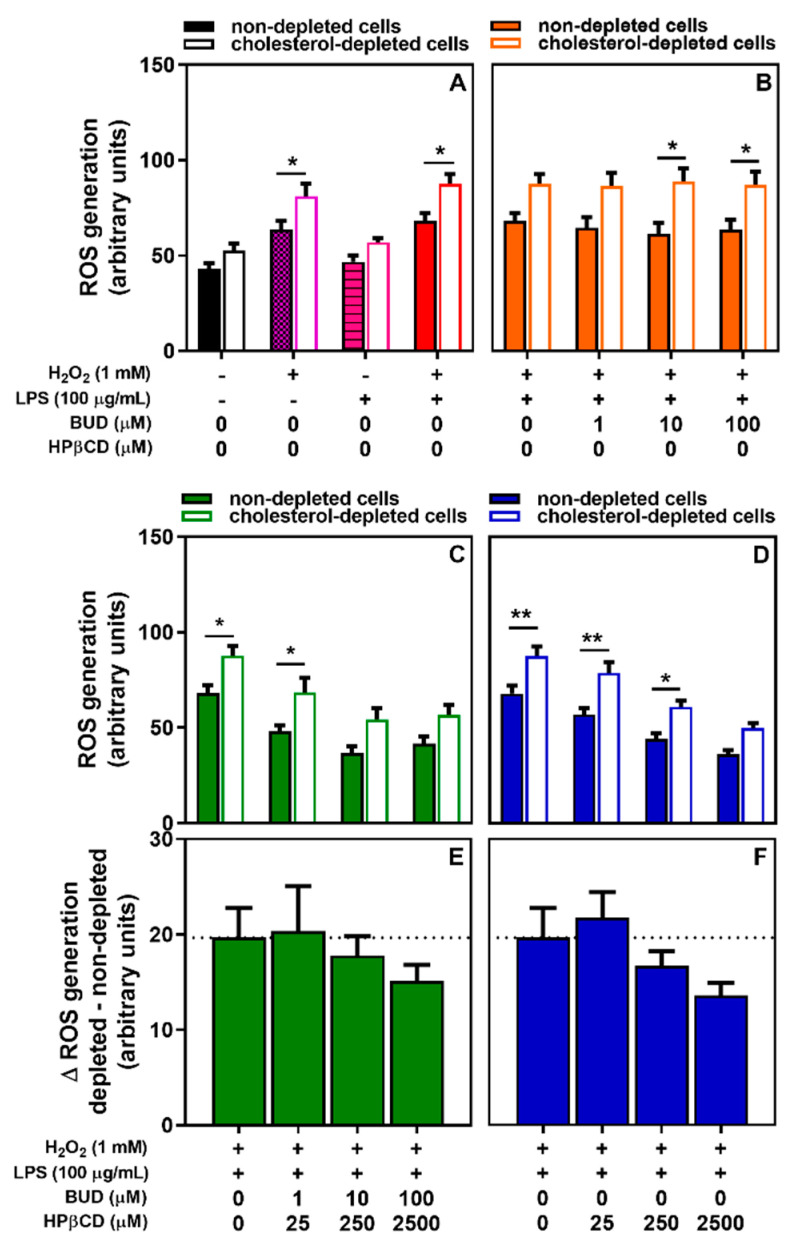
ROS generation in cholesterol-non-depleted or cholesterol-depleted A549 cells after treatment with H_2_O_2_, LPS, or H_2_O_2_ + LPS for 2 h (**A**) and effect of BUD (**B**), BUD:HPβCD complex (**C**) and HPβCD (**D**) on H_2_O_2_ + LPS-induced ROS generation. Panels E and F show the difference (∆) between the effect observed in cholesterol-depleted and -non-depleted cells of BUD:HPβCD complex (**E**) and HPβCD (**F**) on H_2_O_2_ + LPS-induced oxidant generation after treatment for 2 h. Each bar represents the mean ± SEM of 4 independent means of triplicated measures. H_2_O_2_ + LPS-treated bar is the same for each panel. A one-way ANOVA with Dunett post-test was used to compare the mean of each test group in panels (**E**,**F**) with the mean of the H_2_O_2_ + LPS-treated group. A two-way ANOVA with Tukey multiple comparison post-test was used to compare the mean of non-depleted group with the mean of cholesterol-depleted group in the same concentration (panels **A**–**D**). The difference was considered significant for a *p*-value < 0.05. (*) and (**) indicate, respectively, *p* < 0.05 and 0.01 between non-depleted and cholesterol-depleted group (panels **A**–**D**).

In conditions where cholesterol was partly depleted (see Figure S1) we observed (Figure 6A) an increase in basal intracellular ROS levels, although non-significant. A greater and significant increase in H_2_O_2_- and H_2_O_2_ + LPS-induced intracellular oxidant generation was also observed, suggesting that cholesterol content plays a role in H_2_O_2_ + LPS-related oxidative signaling in A549 cells.

Compared to non-depleted cells, the ability of the BUD:HPβCD complex (Figure 6C) and HPβCD (Figure 6D) to protect against H_2_O_2_ + LPS-induced ROS production was preserved. Moreover, when the difference between H_2_O_2_ + LPS-induced ROS production in cholesterol-depleted and non-depleted cells was considered, a concentration-dependent increase in this protective effect was observed (Figure 6E,F), although this was non-significant. Again, no matter the cholesterol status of the cells, budesonide did not show any protective effects (Figure 6B). Thus, cholesterol content might influence the antioxidant effect of the BUD:HPβCD complex and HPβCD.

In contrast with cholesterol, sphingomyelin, another major component from raft and also interacting with HPβCD did not play a critical role on neither the oxidant generation induced by H_2_O_2_ + LPS nor on the ability of the BUD:HPβCD complex and HPβCD to protect against H_2_O_2_ + LPS-induced oxidant generation (Figure S2).

#### 2.4.2. Cholesterol Limits the Effects of the BUD:HPβCD Complex and HPβCD in PI3K/Akt Signaling Induced by H_2_O_2_ + LPS

**Figure 7:**
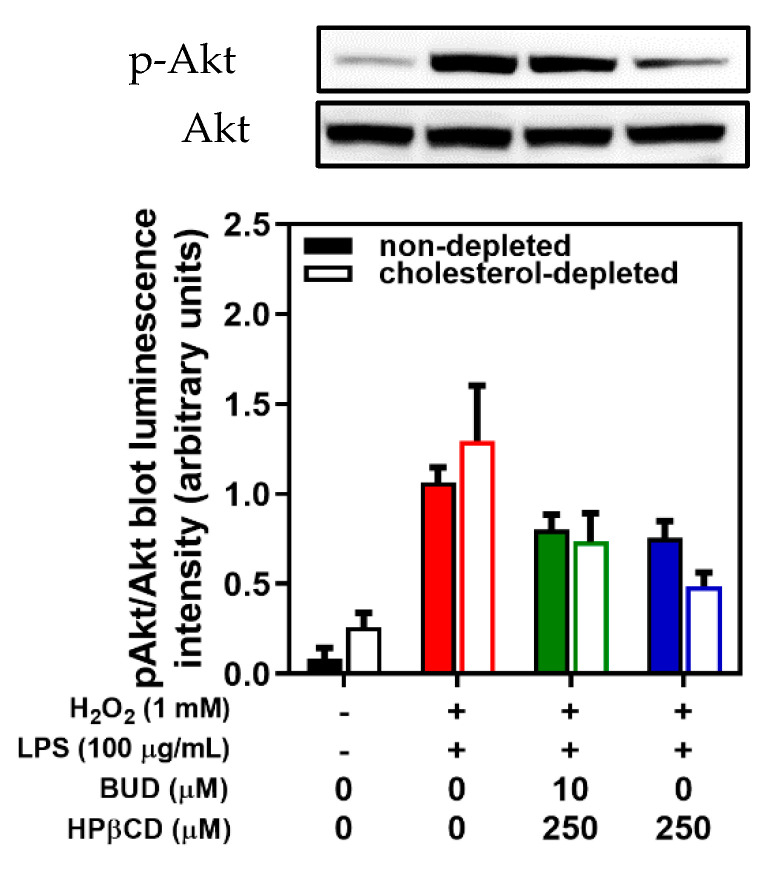
Effect of the BUD:HPβCD complex versus HPβCD on H_2_O_2_ + LPS-induced Akt phosphorylation (p-Akt) in cholesterol-depleted and non-depleted A549 cells after 2 h of incubation with representative blot (cholesterol-depleted cells); each bar represents the mean of 3 independent measures.

Since cholesterol-enriched plasma membrane domains may also play a critical role in the activation of PI3K/Akt signaling [23,25,26], which could be modulated by oxidative stress, the effect of cholesterol depletion on the protective effects of the BUD:HPβCD complex and HPβCD on the phosphorylation of Akt was studied. A decrease in Akt phosphorylation was preserved (Figure 7), without difference in cholesterol-depleted or not depleted cells.

### 2.5. BUD:HPβCD Complex and HPβCD Protect against H_2_O_2_ + LPS-Induced Increase in HDAC2 Phosphorylation in A549 Cells

**Figure 8:**
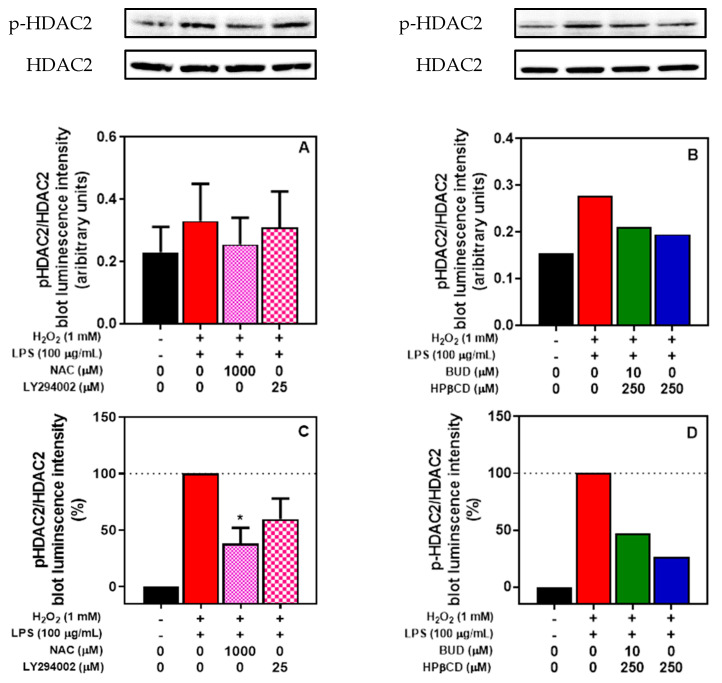
HDAC2 phosphorylation induced by H_2_O_2_ + LPS in A549 cells. Effect of NAC and LY294002 (**A**) and the BUD:HPβCD complex versus HPβCD (**B**) after 2 h of incubation. Data are expressed in absolute values (**A**/**B**; with representative blots) or in relative values (in comparison with the pHDAC2/HDAC2 ration of cells incubated with H_2_O_2_ + LPS; **C**/**D**). HDAC2 phosphorylation was quantified after a Western blot by measuring the proportion of phosphorylated-HDAC2 (p-HDAC2) blot luminescence intensity/total HDAC2 (HDAC2) blot luminescence intensity. Each bar represents the mean of 3 ± SEM or 2 independent measures. A one-way ANOVA with Dunett post-test was used to compare the mean of each test group with the mean of the control group (H_2_O_2_ + LPS-treated group). The difference was considered significant for a *p*-value < 0.05. (*) indicate *p* < 0.05 versus control group.

The increase in PI3K/Akt signaling induced by oxidative stress results in the phosphorylation of HDAC2, a critical step in oxidative stress-related glucocorticoid insensitivity [38,39]. The relationship between oxidative stress and HDAC2 phosphorylation as well as the relationship between the increase in PI3K/Akt signaling and HDAC2 phosphorylation was investigated by using NAC and LY294002, respectively (Figure 8A,C). The treatment of A549 cells with H_2_O_2_ + LPS for 2 h was associated with an increase in phosphorylated HDAC2. Concomitant incubation with NAC or LY294002 was associated with a lower phosphorylation of HDAC2 of approximately 62% (NAC) and 40% (LY294002) (Figure 8A,C). This confirms that H_2_O_2_ + LPS increases HDAC2 phosphorylation through a mechanism involving oxidative stress and PI3K/Akt signaling in A549 cells.

A concomitant incubation with the BUD:HPβCD complex or HPβCD with H_2_O_2_ + LPS was associated with a lower phosphorylation of HDAC2 of approximately 53% (BUD:HPβCD) and 74% (HPβCD) (Figure 8B,D). Thus, the BUD:HPβCD complex and HPβCD inhibited the decrease in HDAC2 activity induced by H_2_O_2_ + LPS treatment in A549 cells with a higher effect induced by HPβCD.

### 2.6. BUD:HPβCD Complex and HPβCD Attenuate H_2_O_2_ + LPS-Induced Inflammatory Response in THP-1 Cells

Since persistent inflammatory response in the lung is a major feature of smoking-induced COPD [31], the anti-inflammatory potential of the BUD:HPβCD complex in comparison with BUD or HPβCD was evaluated. Thus, the effect of the BUD:HPβCD complex, BUD or HPβCD on H_2_O_2_ + LPS-induced IL-8 release in A549 cells and TH-P1 cells was determined. Because A549 cells appeared not sensitive to LPS [40], phorbol myristate acetate-activated THP-1 (A-THP-1) cells [41], a widely used model for human monocytes, which are highly sensitive to LPS treatment, were used. For the sake of comparison, we also treated A549 cells with TNF-α for inflammatory stress.

**Figure 9:**
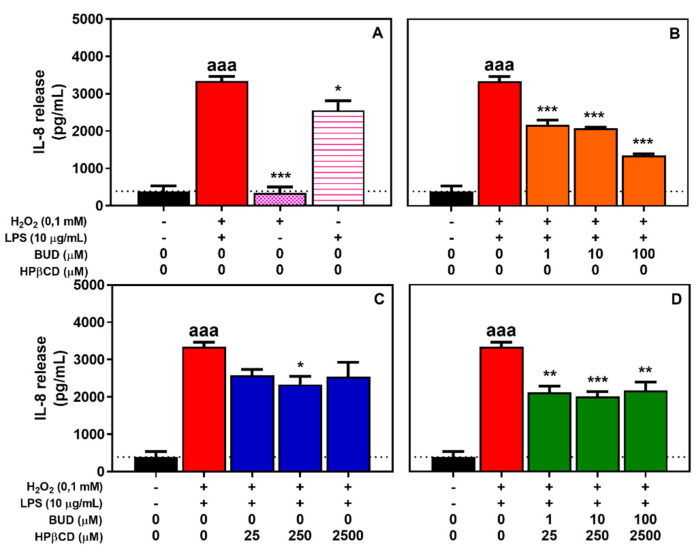
IL-8 release by A-THP-1 cells after treatment with H_2_O_2_, LPS or H_2_O_2_ + LPS for 2 h (**A**) and effect of BUD (**B**), HPβCD (**C**), and BUD:HPβCD complex (**D**) on H_2_O_2_ + LPS-induced IL-8 release. IL-8 release was measured in the extracellular medium by sandwich ELISA. Each bar represents the mean ± SEM of 3 independent means of triplicated measures. A one-way ANOVA with Dunett post-test was used to compare the mean of each test group with the mean of untreated group or H_2_O_2_ + LPS-treated group. The difference was considered significant for a *p*-value < 0.05; (aaa), indicate *p* < 0.001 versus untreated group; (*), (**), and (***) correspond to *p* < 0.05, 0.01 and 0.001 versus H_2_O_2_ + LPS-treated group, respectively.

First, the incubation of A-THP-1 cells with H_2_O_2_ + LPS for 2 h was significantly associated with an 8.6-fold increase in IL-8 release (Figure 9A). Treatment with LPS alone induced a significant 6.6-fold increase, whereas H_2_O_2_ alone did not induce any effects on IL-8 expression [41].

Concomitant incubation of the BUD:HPβCD complex with H_2_O_2_ + LPS was associated with a lower release of IL-8 of approximately 45% no matter the concentration of the BUD:HPβCD complex used (Figure 9D). In the presence of BUD, a decrease in of IL-8 release was also observed. The effect was similar with that induced by the BUD:HPβCD complex (budesonide 1 and 10 µM), but higher at higher budesonide concentration (100 µM) (67%) (Figure 9B). The presence of HPβCD was also associated with a non-dependent dose-type decrease in IL-8 release. The effect was slightly lower as compared to BUD and the BUD:HPβCD complex (approximately 34%) (Figure 9C). These results suggest that the BUD:HPβCD complex and BUD have similar anti-inflammatory properties, except at high concentrations at which budesonide was more efficient.

**Figure 10:**
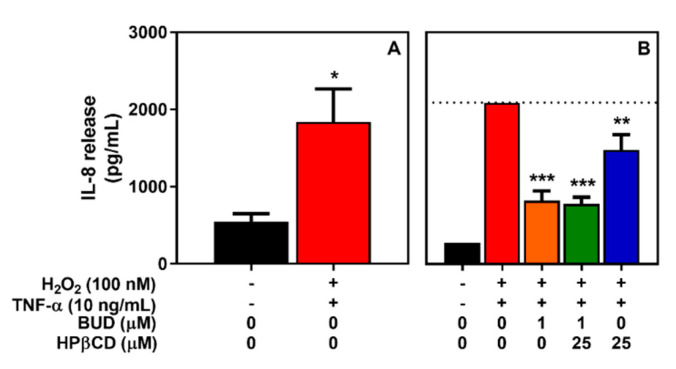
IL-8 release by A549 cells after treatment with H_2_O_2_ + TNF-α for 2 h (**A**) and effect of the BUD:HPβCD complex versus BUD and HPβCD on H_2_O_2_ + TNF-α-induced IL-8 release (**B**). IL-8 release was measured in the extracellular medium by sandwich ELISA. Results on panel B were normalized relative to untreated cells (0%) and H_2_O_2_ + TNF-α-treated cells (100%). Each bar represents the mean ± SEM of 3 independent means of triplicated measures. (*), (**), and (***), indicate, respectively, *p* < 0.05, 0.01, and 0.001 versus non-treated cells (**A**) or H_2_O_2_+LPS-treated cells (**B**).

A similar effect on IL-8 release was reported when comparing the effect of TNFα on A549 cells with the effect LPS on A-THP1. An anti-inflammatory potential of BUD (1 µM) and the BUD:HPβCD complex (1:25 µM) was observed with a lower potential of HPβCD to decrease IL-8 release (Figure 10).

**Figure 11:**
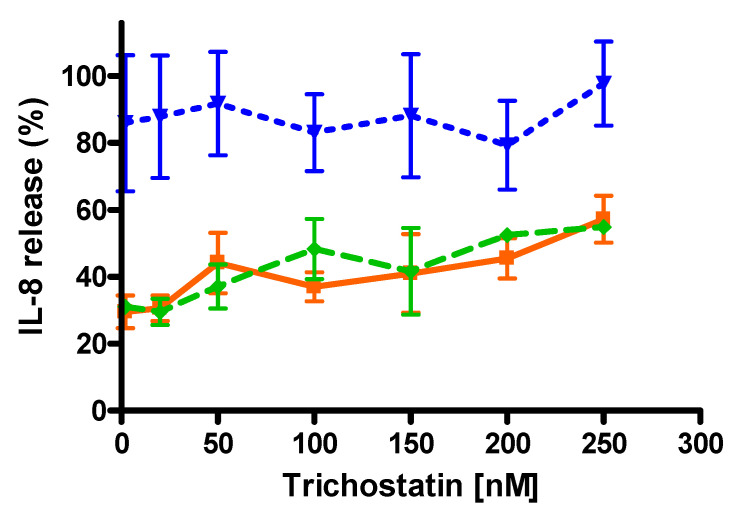
Percentage of IL-8 released after A549 cells pretreatment for 30 min with trichostatin (TSA) and incubation for 2 h with TNF-α in presence of BUD:HPβCD complex, BUD or HPβCD. Results are expressed in percentage of IL-8 released. 100% corresponds to cells preincubated for 30 min with TSA and incubated for 2 h with TNF-α only. IL-8 release was measured in the extracellular medium by sandwich ELISA. Data are from 3 independent experiments in triplicates. ▼HPβCD; ♦ BUD:HPβCD; ■ BUD.

As HDAC2 is recruited by the activated glucocorticoid receptor to repress the transcription of proinflammatory genes [42] and to study the potential role of HDAC2 in the protection afforded by the BUD:HPβCD complex in comparison with BUD or HPβCD on IL-8 release, IL-8 release induced by TNF-α in conditions where cells were preincubated with or without trichostatin, a pharmacological HDAC2 inhibitor [43], was measured. We pretreated for 30 min A549 cells with increasing concentrations of trichostatin (0–250 nM) and determined IL-8 release after incubation for 2 h of cells with TNF-α (20 ng/mL) and BUD:HPβCD complex or BUD or HPβCD (Figure 11).

IL-8 release induced by TNFα was markedly reduced (around 70%) by BUD and BUD:HPβCD whereas HPβCD alone did not shown any effect or a very slight effect. When trichostatin was used in preincubation to inhibit the HDAC2 activity, the IL-8 release was increased, in a dose-dependent fashion in the presence of BUD or BUD:HPβCD. BUD:HPβCD failed to improve the response of glucocorticoids in the condition of HDAC2 inhibition. Again, no or a very slight effect was observed with HPβCD (Figure 11).

## 3. Discussion

In animal models, Dufour et al. [12] suggested that budesonide (BUD) complexed with 2-hydroxypropyl-β-cyclodextrin (HPβCD) might be an alternative to BUD alone in the treatment of smoking-induced COPD. The current study was designed to characterize the effect of the BUD:HPβCD complex on the response of human alveolar epithelial cells (A549) or human monocytes (A-THP1) to a mix of hydrogen peroxide and lipopolysaccharides (H_2_O_2_ + LPSs) mimicking stressful effects including those from cigarette smoke [28,44,45] or from environmental toxicants. We characterized the effect of the BUD:HPβCD complex on (i) ROS generation (oxidative stress), (ii) Akt phosphorylation (PI3K/Akt signaling activation), (iii) HDAC2 phosphorylation (HDAC2 inhibition of activity), and (iv) IL-8 release (inflammatory response) in comparison with the effects induced by BUD or HPβCD.

We demonstrated the protective effect afforded by BUD:HPβCD against cytotoxicity and ROS generation induced by oxidative and inflammatory stress in comparison with BUD. The effect observed for BUD:HPBCD was comparable to that observed with HPBCD and might be limited by cholesterol. We also demonstrated (i) the involvement of the canonical molecular pathway including ROS generation, decrease in PI3K/Akt activation, decrease in HDAC2 activity and insensitivity to glucocorticoid in the effect induced by BUD:HPβCD, (ii) maintenance of IL-8 decrease with BUD:HPβCD—even BUD at a high concentration (100 µM) induced a slightly higher effect—and (iii) the absence of improvement in glucocorticoid insensitivity with BUD:HPβCD in comparison with BUD, in conditions where HDAC2 was inhibited.

Improvement of cell viability after oxidative and inflammatory stress induced by BUD:HPβCD is likely due to HPβCD and linked to a decrease in ROS generation. The literature has reported that cyclodextrins, including HPβCD, may improve the toxicological profile of drugs by complexing them [46,47]. Additionally, the antioxidant potential of HPβCD has been reported. Anraku et al. [48] showed HPβCD remove pro-oxidants such as uremic toxins from the blood in a rat model of chronic renal failure. Zimmer et al. [49] showed that HPβCD decreases aortic ROS generation in a mouse model of atherosclerosis. Other reports reviewed by López-Nicolás et al. [50] described HPβCD as a protective agent of lipophilic nutrients and antioxidants against oxidation in foods. The demonstration of HPβCD’s antioxidant potential is interesting given the major role played by oxidative stress in numerous pathologies including COPD [51]. Here, the molecular mechanism leading to a decrease in ROS is still unclear but a direct effect through the interaction of HPβCD with H_2_O_2_ (Figure S3) is unlikely.

An indirect effect through changes in biophysical membrane properties could be suggested as an alternative explanation. We initially suggested that membrane cholesterol would play a major role in the occurrence of BUD:HPβCD-related cytoprotective effects. It has been extensively demonstrated that βCDs, including HPβCD, can interact with lipid membranes, and change membrane biophysical properties [17,35] closely related to signal transduction. This agrees with our previous experiments on giant unilamellar vesicles (GUVs), since we demonstrated BUD:HPβCD and HPβCD disrupted the liquid-disordered/liquid-ordered (Ld/Lo) phase separation observed in the presence of cholesterol for the benefit of the Ld phase, a process hindered in the presence of cholesterol [27]. Here, we observed an increase in BUD:HPβCD-related antioxidant effects in cholesterol-depleted cells suggesting that cholesterol might hinder ROS generation. The BUD:HPβCD-related antioxidant effect was preserved and even increased in cells partially depleted in cholesterol (50% cholesterol depletion after 30 min exposition to MβCD at 5 mM; no or very small cholesterol depletion induced by HPβCD for 2 h at the highest concentrations used in this work; Figure S4). Extracellular mechanisms are unlikely since we observed (i) no cellular uptake of HPβCD over the entire incubation period (Figure S5), and (ii) no neutralization of extracellular signals potentially responsible for oxidative stress, namely H_2_O_2_ and free radicals (Figure S3). BUD:HPβCD and HPβCD-related cytoprotective effects could be seen as a membrane-mediated mechanism involving membrane lipid disorganization with limited lipid extraction after 2 h (cholesterol extraction induced by BUD:HPβCD or HPβCD reached 18% and 12%, respectively, while no cholesterol extraction in cholesterol-depleted cells was observed (Figure S4)), agreeing with the work of Lopez et al. [52].

One remaining question is the cross-talk between the antioxidant effect and inhibiting effect on oxidant-induced PI3K/Akt signaling. NAC and Vit C concentrations that inhibit more than 75% of ROS generation after 2 h of incubation (Figure S6) showed a protective effect against H_2_O_2_ + LPS-induced increase in PI3K/Akt signaling of about 36% (NAC) and ~65% (Vit C). The effect was not related to the effect of LPS which might induce an increase in PI3K/Akt signaling [53,54] since we observed that H_2_O_2_ + LPS-induced increase in PI3K/Akt signaling was almost exclusively associated with the presence of H_2_O_2_ (Figure S7). The activity of endogenous antioxidant enzymes GSH peroxidase against H_2_O_2_ [55], difference in the location within the bilayer between the effect induced by BUD:HPBCD and the location of enzymes involved in ROS generation or PI3K/Akt activation could be also involved.

Focusing on the final attempt for BUD:HPBCD, meaning its ability to decrease the release of inflammatory cytokines after oxidant and inflammatory stress, we could have expected a higher anti-inflammatory effect of the BUD:HPβCD complex compared to the BUD alone. Dufour et al. [12] in a murine asthma model showed that similar anti-inflammatory effects could be obtained with a 2.5-fold lower BUD concentration when given as a complex with HPβCD. Zimmer et al. [49] reported anti-inflammatory effects of HPβCD in vivo in a mice model of atherosclerosis. At the cellular level, George et al. [56] assumed an anti-inflammatory property of HPβCD after showing that its presence along with plasticized poly(vinyl chloride) (PVC) reduced LPS-induced TNF-α expression in human monocyte-like U937 cells while PVC alone had no effect. Matassoli et al. [57] showed that HPβCD can inhibit LPS-induced TNF-α secretion in primary human monocytes. The higher effect on reduction of IL-8 release induced by BUD at a high concentration (100 µM) as compared to the effect of BUD:HPβCD could be linked to an inflammatory effect observed at high doses of HPβCD [58]. Cell-type cellular components of inflammation [59] and changes in the release of BUD from HPβCD hydrophobic cavity, depending upon the concentrations, could also play a role.

Lastly, in a potential translational perspective, the design of studies and concentrations have to be questioned. First, cells were exposed with BUD and H_2_O_2_ + LPS at the same time, meaning that BUD had time to prevent the inflammatory response before the decrease in glucocorticoid sensitivity induced by oxidative stress could take place. We reproduced experiments by changing the time course and by preincubating cells for 30 min with the oxidant and inflammatory stress before the incubation of cells with BUD:HPβCD for 2 h. No differences were observed. Another critical parameter would be the equilibrium between the free and bound forms of BUD or HPBCD [60]. In the presence of a lipophilic membrane, drug partitioning from the complex into the membranes can occur, promoting drug release from the CD hydrophobic cavity. The latter point agrees with our work. Indeed, we showed that in pure phospholipid monolayers there is an increase in membrane surface pressure with the BUD:HPβCD complex but not with HPβCD. This increase is usually associated with the insertion of a molecule within the monolayer. Since the only difference between HPβCD and the BUD:HPβCD complex is the presence of BUD, we could assume that BUD was inserted within the membrane. The critical importance of the equilibrium between free and complexed budesonide was also evidenced when we determined the effect of a mix of BUD and HPβCD on IL-8 release for cells treated with increasing concentrations of trichostatin, a pharmacological inhibitor of HDAC2. The protective effect against IL-8 release of the mixture was higher than that afforded by the complex (Figure S8).

Second, BUD concentrations and/or amount of oxidant and inflammatory stressors used are relevant for patho-physio-logical conditions. BUD dry powder for inhalation (Pulmicort^®^), was recommended for COPD patient administration—up to 1000 μg/day on average. If we assume that about 30% of the nominal dose inhaled with a dry powder inhaler might reach the lungs [61,62], therefore 300 μg of BUD dry powder in Pulmicort^®^ administered in patients might reach the lungs. If the 300 μg of BUD will disperse in the lung lining fluid (20–40 mL in a human of 70 kg) [63], then the pulmonary BUD concentration could be approximately 17–35 μM, which is in the range of concentrations used in our study (1–100 μM). However, we must remain cautious since the amount of BUD deposited in the lung is difficult to predict. The question of the relevance of the quantity of H_2_O_2_ + LPS is also raised. Here again, it appears difficult to properly assess the exposition of alveolar cells to H_2_O_2_ and LPS during smoking—e.g., many factors should be considered, such as the frequency of smoking, the number and type of cigarettes smoked per day, the duration of smoking, the distribution of smoke in the lungs, the half-life of each molecular species generated in cigarette smoke, their own biodisponibility, and so on. Nakayama et al. [28] and Hasday et al. [45], respectively, reported that extract amounts of H_2_O_2_ ranging from 500 nmol to 4 μmol of H_2_O_2_ per cigarette and 6 to 9 μg of active LPS per gram of a cigarette can be extracted. The amount of H_2_O_2_ used in this work appears less important (up to 200 nmol in 200 μL), whereas the amount of LPS appears in the same range (up to 10 μg in 100 μL).

In conclusion, we demonstrated the anticytotoxic, antioxidant, anti-inflammatory properties of the BUD:HPβCD complex with protective activity against PI3K/Akt signaling activation and HDAC2 inhibition induced by oxidative stress. The antioxidant and anticytotoxic properties appeared essentially due to HPβCD while the anti-inflammatory properties appeared mainly to be due to BUD. Further investigations are clearly needed for a more complete view of the potential of the BUD:HPβCD complex in other relevant models of oxidative stress-induced glucocorticoid insensitivity in vitro or in vivo.

## 4. Material and Methods

### 4.1. Material

A549 (ATCC^®^ CCL185™) and THP-1 (ATCC^®^ TIB-202™) cells were purchased from the American Type Culture Collection (Manassas, VA, USA). HPβCD was obtained from Roquette, Lestrem, France. H_2_O_2_, Lipopolysaccharides (LPSs), Budesonide (BUD), Phorbol Myristate Acetate (PMA), MβCD, sphingomyelinase from Bacillus cereus, *N*-acetyl-l-cysteine (NAC), 2,2-Diphenyl-1-picrylhydrazyl (DPPH•), and l-ascorbic-acid (vitamin C) were ordered from Sigma-Aldrich (Saint Louis, MO, USA). A Cytotoxicity Detection KitPLUS (LDH) was ordered from Roche (Mannheim, Germany) and HOECHST^®^ 33,342 staining solution from Life technologies (Eugene, OR, USA). LY294002 was ordered from Gibco (Camarillo, CA, USA). Phospho-Akt (Ser473) (D9E) XP^®^ and Akt rabbit monoclonal antibodies were obtained from Cell Signaling Technology^®^ (Beverly, MA, USA). β-actin (C4), mouse IgGκ light chain binding protein (m-IgGκ BP) conjugated to horseradish peroxidase (HRP) and mouse antirabbit IgG-HRP monoclonal antibodies were obtained from Santa Cruz Technology (Dallas, TX, USA). Horseradish Peroxidase (HRP) was ordered from Thermo Scientific^TM^ (Rockford, IL, USA). Anti-HDAC2 (Ab-394) and Anti-phospho-HDAC2 (pSer394) antibodies produced in rabbit were ordered from Sigma-Aldrich (Saint Louis, MO, USA). Phenolsulfonphtalein (phenol red) was obtained from Merck (Darmstadt, Germany). A Human IL-8/CXCL8 DuoSet ELISA kit was obtained from R&D systems (Minneapolis, MN, USA). Trimethylsilyl-3-propionide acid-*d4* (TMSP) and deuterium oxide (99.96% D) were purchased from Eurisotop (Gif-sur-Yvette, France). Certified maleic acid and phosphate buffer powder were provided by Sigma-Aldrich (Karlsruhe, Germany). NMR measurements were recorded on a Bruker Avance spectrometer operating at 500.13 MHz for the proton signal acquisition and equipped with a 5-mm TCI cryoprobe with a Z-gradient.

### 4.2. BUD:HPβCD Complex Stock Solution Preparation and Characterization

We adapted the method of Dufour et al. [64]. The BUD:HPβCD complex stock solutions were prepared by adding 200 mM HPβCD (molar substitution = 0.64) in deionized water to BUD powder at a final concentration of 8.13 mM (BUD:HPβCD 1:25 molar ratio). The solution was then thoroughly mixed for 1 h 30 min (13,500 rpm) with a T25 basic Ultra-Turrax^®^ homogenizer from IKA (Staufen, Germany) and filtered (0.22-µm filter unit). BUD and HPβCD were quantified in the solution obtained by HPLC-UV and ^1^H-NMR, respectively, and checked for complexation as described by Dufour et al. [64]. Solutions were stored at 4 °C and renewed every 2 months.

### 4.3. Cell Handling

A549 [40] and THP-1 [41] cells were grown in DMEM (1X) and RPMI-1640 medium (1X) (Gibco, Paisley, UK), respectively, and were both supplemented with FBS (10%) and penicillin-streptomycin (1%) (Gibco, Grand Island, NY, USA) at 37 °C in a 5% CO_2_ humidified atmosphere. Sub-cultures were performed according to the manufacturer’s instructions. To activate THP-1 in macrophage-like cells (A-THP-1), cells were resuspended in fresh media, and phorbol 12-myristate 13-acetate (PMA) was added (final concentration 200 µg/L) to the THP-1-containing medium [65]. A549 and THP-1 cells were then seeded in culture microplates, dishes or flasks depending on the experiment and incubated until sub-confluent (A549, ~80%) or 24 h (THP-1) at 37 °C in a 5% CO_2_ humidified atmosphere. For experiments, test molecules were dissolved in 1% FBS-supplemented medium, unless otherwise mentioned. When prior cell cholesterol or sphingomyelin depletion was required, cells were preincubated for 30 min with 5 mM methyl-β-cyclodextrin (MβCD) or 50 mU/mL of sphingomyelinase from *Bacillus cereus* [66] in 1% FBS-supplemented medium, respectively.

### 4.4. Cytotoxicity Studies

#### 4.4.1. Lactate Dehydrogenase Assay

A549 cells in 96-well plates were incubated with increasing concentrations of BUD, HPβCD, or BUD:HPβCD complex, or with H_2_O_2_ + LPS with/without increasing concentrations of BUD, HPβCD, or BUD:HPβCD complex. The activity of lactate dehydrogenase (LDH) released by non-viable cells in the supernatant was quantified using the Cytotoxicity Detection KitPLUS (LDH) from Roche (Mannheim, Germany) according to the manufacturer’s instructions.

#### 4.4.2. HOECHST Nuclear Staining

A549 cells in ibiTreat µ-slides 2 wells from ibidi (Martinsried, Germany) were incubated with H_2_O_2_ + LPS with/without BUD, HPβCD, or BUD:HPβCD complex. Cells were then washed with PBS, covered with a 2000-fold dilution of HOECHST^®^ 33,342 staining solution (Life technologies, Eugene, OR, USA) in PBS, and incubated 5 min at room temperature protected from light. Cells were then washed with PBS and imaged with a fluorescence microscope (λ_ex_/_em_ = 350/461, DAPI filter set). Cells with bright and/or fragmented nuclei were considered apoptotic. The proportion of apoptotic cells was calculated from a total cell count of 400/well. H_2_O_2_ + LPS concentrations were those preselected for LDH assay.

### 4.5. DCF Assay for Determining ROS Generation

We adapted the method of Wang and Joseph [67]. Briefly, A549 cells in 96-well plates were incubated for 30 min with 10 or 50 µM membrane-permeant and non-fluorescent 2′,7′-dichlorofluorescein diacetate (DCFDA) (Sigma-Aldrich, Saint Louis, MO, USA), which was deacetylated by non-specific intracellular esterases into the membrane-impermeant and non-fluorescent DCFH_2_. Cells were then washed with Hank’s balanced salt solution (HBSS) and incubated with H_2_O_2_ + LPS with/without BUD, HPβCD, or BUD:HPβCD complex in HBSS. Oxidative stress was evaluated through the measure of the fluorescence of DCF resulting from the oxidation of DCFH_2_ by intracellular oxidants (λ_ex_ = 490 nm; λ_em_ = 523 nm).

### 4.6. Evaluation of Protein Quantity by Western Blotting

A549 cells in 6-well plates or 60 × 15 mm culture dishes were incubated with H_2_O_2_ + LPS with/without BUD, HPβCD, or BUD:HPβCD complex. After incubation, cells were washed with ice-cold PBS and scraped off with a cold scraper in the presence of ice-cold RIPA or Biovision’s cell lysis buffer supplemented with protease and phosphatase inhibitor cocktails. Detached cells in lysis buffer were then incubated for 30 min at 4 °C with agitation in a 2-mL microcentrifuge tube and centrifuged for 10 min (10,000× *g*, 4 °C). The supernatant (whole-cell lysate) was stored at −80 °C at least overnight. A quantity of 30 µg of proteins per sample was mixed with 1X NuPAGE LDS sample buffer and 1X NuPAGE sample reducing agent (Thermo Scientific^TM^, Carlsbad, CA, USA) and heated for 10 min at 70 °C. Samples were then electrophoresed on precasted NuPAGE Bis-Tris gels in the presence of MOPS (3-(N-morpholino)propanesulfonic acid) running buffer 1X, transferred to PVDF (Polyvinylidene difluoride) transfer membranes (Thermo Scientific^TM^, Rockford, IL, USA) in the presence of NuPAGE transfer buffer 1X (Thermo Scientific^TM^, Carlsbad, CA, USA) and blocked for 1 h in 5% non-fat dry milk in 20 mL of tris-buffered saline 1X containing 0.05% Tween 20 (TBS-T). Membranes were incubated overnight at 4 °C with primary antibodies with gentle agitation, washed 3 times with TBS-T, then incubated for 1 h at room temperature with the appropriated HRP-conjugated secondary antibodies. The manufacturer’s recommendations were followed for antibody dilutions. After washing 3 times with TBS-T, blots were revealed using the SuperSignal West Pico Chemiluminescent Substrate (Thermo Scientific^TM^, Rockford, IL, USA), the Fusion Pulse 7 apparatus and Fusion Capt Advance Pulse 7 software. To reveal proteins with similar migration profiles, membranes were washed in TBS-T after the first reveal, and antibodies were stripped with a 10-min bath in Restore^TM^ Western Blot Stripping Buffer (Thermo Scientific^TM^, Rockford, IL, USA) and washed again with TBS-T. Then, the Western blot protocol was repeated from the block for 1 h in 5% non-fat dry milk in 20 mL of TBS-T.

### 4.7. Evaluation of Inflammatory Cytokine (IL-8) Expression by Sandwich ELISA

A-THP-1 cells in 96-well plates were incubated with H_2_O_2_ + LPS with/without BUD, HPβCD, or BUD:HPβCD complex. A 4-fold dilution of the supernatant in RPMI-1640 medium was stored overnight at −80 °C. IL-8 cytokine levels in diluted supernatant were quantified using the Human IL-8/CXCL8 DuoSet ELISA kit (R&D systems, Minneapolis, MN, USA) according to the manufacturer’s instructions.

### 4.8. Data Analysis

GraphPad Prism^®^ (version 4.03 for Windows, GraphPad Prism Software, San Diego, CA, USA) was used for graphic illustrations and statistical analysis. The statistical tests used to study the significance of the results are described in the captions of the corresponding figures.

## Supplementary Materials

The following are available online. Figure S1: Cholesterol content in A549 cells untreated (control), incubated with methyl-β-cyclodextrin (MβCD) for 30 min, and incubated with methyl-β-cyclodextrin (MβCD) for 30 min and thereafter in medium for 2 h. Figure S2: Effect of the BUD: HPβCD complex (green) and HPβCD (blue) on oxidant generation induced by H_2_O_2_ + LPS after treatment for 2 h in non-depleted and sphingomyelin-depleted A549 cells. Figure S3: Effect of the BUD:HPβCD complex versus HPβCD on H_2_O_2_ (**A**) and 2,2-Diphenyl-1-picrylhydrazyl (DPPH•) (**B**) after 25 min (**A**) and 1 h of incubation (**B**). Figure S4: Cholesterol content in A549 cells incubated with the BUD:HPβCD complex, HPβCD or BUD for 2 h. Figure S5: HPβCD relative quantity in A549 extracellular medium after 2 h of incubation with the BUD:HPβCD complex (green bars) and HPβCD (blue bars). HPβCD was quantified using proton nuclear magnetic resonance spectroscopy. Figure S6: Oxidant generation kinetic in A549 cells after treatment with H_2_O_2_ + LPS (1 mM + 100 μg/mL) for 6 h and effect of *N*-acetyl-l-cysteine (NAC) and Vit C on H_2_O_2_ + LPS-induced oxidant generation. Figure S7: Akt phosphorylation induced by H_2_O_2_ + LPS, H_2_O_2_ and LPS in A549 cells after 2 h of incubation. Figure S8: Percentage of IL-8 released after A 549 cells pretreatment for 30 min with trichostatin (TSA) and incubation for 2 h with TNF-α in presence of BUD + HPβCD complex, BUD or HPβCD [68,69,70,71].
