# Supplementary material for: The Budesonide-Hydroxypropyl-β-Cyclodextrin Complex Attenuates ROS Generation, IL-8 Release and Cell Death Induced by Oxidant and Inflammatory Stress. Study on A549 and A-THP-1 Cells
Source: Molecules. 2020 Oct 22;25(21):4882. doi: 10.3390/molecules25214882 (PMC7660049; doi:10.3390/molecules25214882)
Supplement: Supplementary file 1 [file molecules-25-04882-s001.pdf]

Supplementary Material

Cholesterol content in A549 cells untreated (control), incubated with methyl-β-cyclodextrin (MβCD) for 30 min, and incubated with methyl-β-cyclodextrin (MβCD) for 30 min and thereafter in medium for 2 hours

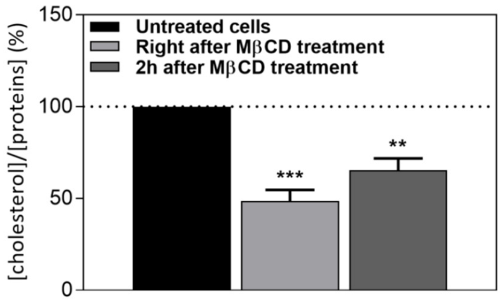

Figure S1. Cholesterol content in A549 cells untreated (control), incubated with methyl-β-cyclodextrin (MβCD) for 30 min, and incubated with methyl-β-cyclodextrin (MβCD) for 30 min and thereafter in medium for 2 hours. Cholesterol content was measured with the amplex red cholesterol assay kit from Invitrogen and normalized with protein concentrations. The results were normalized relative to untreated cells (100%). Each bar represents the mean ± SEM of 3 independent means of triplicated measures (N = 3). (**) and (***), respectively, indicate $p < 0.01$, and 0.001 versus untreated cells.

Effect of sphingomyelin depletion on H_2O_2 + LPS-induced oxidative stress and on the effect of the BUD:HPβCD complex or HPβCD

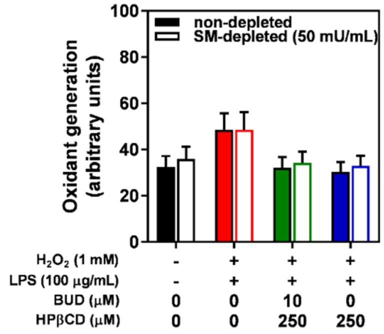

Figure S2. Effect of the BUD: HPβCD complex (green) and HPβCD (blue) on oxidant generation induced by H_2O_2 + LPS after treatment for 2 h in non-depleted and sphingomyelin-depleted A549 cells. Each bar represents the mean ± SEM of 6 independent means of triplicated measures.

Effect of the BUD:HPβCD complex on extracellular oxidative signals

Evaluation of H_2O_2 Neutralization by the Phenol Red Assay

We adapted the method of Pick and Keisari [68] modified by Bahorun et al. [69], based on the H_2O_2-dependent HRP-mediated oxidation of phenol red resulting in a change in its absorption

spectrum. A 0.1 M concentration of potassium phosphate buffer at pH 7 was prepared by mixing 61.5 mL of 1 M K_2HPO_4 and 38.5 mL of 1 M KH_2PO_4 in deionized water and bringing the volume to 1 L with deionized water. We added 100 μL of HPβCD or budesonide (BUD):HPβCD complex solutions or vitamin C and 100 μL of 30 μM H_2O_2, both in 0.1 M potassium phosphate buffer, to 800 μL of 100 mM NaCl in 0.1 M potassium phosphate buffer and incubated the solution for 10 min at 37°C. Then, we added 1 mL of 0.2 mg/mL phenol red dye with 0.1 mg/mL Horseradish Peroxidase (HRP) in 0.1 M phosphate buffer. After 15 min, we added 50 μL of 1 M NaOH, and the absorbance was immediately read at 610 nm.

Evaluation of Free Radical Neutralization by DPPH• Assay

We adapted the method developed by Brand-Williams et al. [70]. DPPH• is a stable free radical that exhibits a deep violet color in methanol solution, with an absorption band centered at 515 nm. Its reduction, resulting from the pairing of its free electron, causes a reduction in its absorbance at 515 nm. We added 100 μL per well of HPβCD, BUD:HPβCD complex or vitamin C in methanol in triplicate in 96-well plates followed by the addition of 100 μL per well of 2,2-Diphenyl-1-picrylhydrazyl (DPPH•) (final concentration of 100 μM) in methanol. The absorbance at 515 nm was immediately read after addition of DPPH• and at 10-min intervals for 1 h at 30°C.

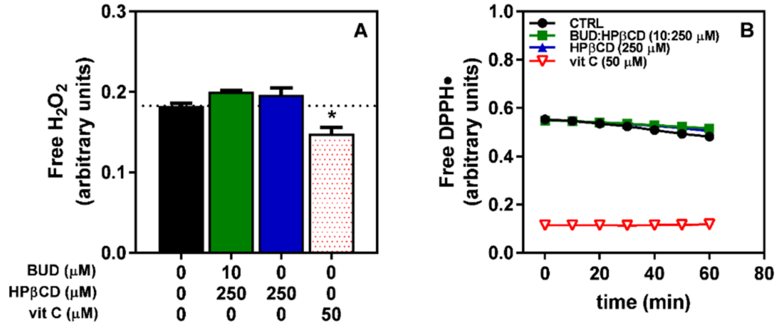

Figure S3. Effect of the BUD:HPβCD complex versus HPβCD on H_2O_2 (A) and 2,2-Diphenyl-1-picrylhydrazyl (DPPH•) (B) after 25 min (A) and 1 h of incubation (B). H_2O_2 neutralization was evaluated by measuring the change in absorbance of phenol red induced by oxidation by Horseradish Peroxidase (HRP), which uses H_2O_2 as a substrate. DPPH• neutralization was evaluated by monitoring the decrease in absorbance at 515 nm along with the level of reduction. Each bar represents the mean ± SEM of 2 independent means of six repeated measures (A). Each point represents the mean of quadruplicated measures (B).

Cholesterol content in A549 cells incubated with the BUD:HPβCD complex, HPβCD or BUD for 2h

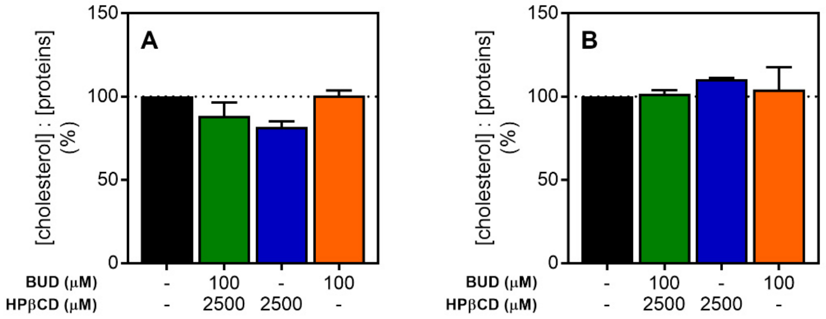

Figure S4. Cholesterol content in A549 cells incubated with the BUD:HPβCD complex, HPβCD or BUD for 2h. A549 cells were preincubated (B) or not (A) with methyl-β-cyclodextrin (5 mM) for 30 min. Cholesterol content was measured with the amplex red cholesterol assay kit from Invitrogen and normalized with protein concentrations. The means in panels A and B come from 2 independent set of experimental data. A one-way ANOVA with Dunett post-test was used to compare the mean of each test group with the mean of the control group in each panel (untreated group). The results were normalized relative to untreated cells (100%). Each bar represents the mean ± SEM of 2 (B) or 3 (A) independent means of triplicated measures (N = 3).

Cellular uptake of HPβCD

Evaluation of HPβCD Cellular Uptake by Proton Nuclear Magnetic Resonance (^{1}H NMR) Spectroscopy

To further question the possible role of the plasma membrane, the first barrier encountered by HPβCD or BUD:HPBCD, in the protection induced by BUD:HPβCD or HPβCD against the oxidative effects resulting from cell incubation with H_2O_2 + LPS for mimicking tobacco smoke, we tried to evidence changes in HPBCD in intra- or extracellular media. We failed to evidence HPβCD in the intracellular medium and we did not observe any change in HPβCD relative quantity in the extracellular medium upon 2 h of incubation of A549 cells with the BUD:HPβCD complex or HPβCD (Figure S3). In addition, we looked for a potential neutralization of H_2O_2 and DPPH• in the extracellular medium. Both mimic free radicals potentially released by cells undergoing oxidative stress [28]. In our hands, they were not neutralized by the BUD:HPβCD complex or by HPβCD (Figure S4). Together, these results suggest that the antioxidant effects of the BUD:HPβCD complex/HPβCD may not result from intracellular mechanisms or the neutralization of oxidative signals within the extracellular environment, supporting the idea of a membrane mechanism.

Intracellular analysis: A549 cells in a 75-cm^2 filter cap cell culture flask were incubated for 2 h with HPβCD or the BUD:HPβCD complex in Hank's balanced salt solution (HBSS). After incubation, cells were washed with HBSS and detached by trypsinization. Detached cells were harvested in 15-mL plastic tubes with ice-cold 10% FBS-supplemented DMEM to inactivate trypsin. In total, 5 x 10^6 cells in solution were taken from each sample, centrifuged (900 rpm, 10 min) in 15-mL plastic tubes and washed twice with ice-cold HBSS (first wash followed by 10-min centrifugation at 900 rpm of samples in 15-mL plastic tubes, second wash followed by 1-min centrifugation at 11000 rpm of samples in 2-mL plastic tubes). The dried pellet was stored overnight at at least -80°C. Cell extracts were then lysed for ^{1}H NMR analysis in accordance with a procedure by Matheus et al. [71] in D_2O buffer. The obtained lysate was then centrifuged to eliminate membranes and cell debris, and 650 µL of the supernatant containing intracellular medium was collected and then supplemented with 2 µL of a 10 mg/mL 3-trimethylsilyl propionic acid (TMSP) deuterium oxide (D_2O) solution and 50 µL of a 35 mM solution of maleic acid. The solution was distributed into 5-mm tubes for NMR measurement, and the ^{1}H NMR spectra were acquired using a CPMG relaxation-editing sequence with presaturation. The CPMG experiment used an RD-90°-(t-180°-t)n-acquire sequence with a relaxation delay (RD) of 4 s, a spin echo delay (t) of 300 µs and a number of loops (n) of 128. The water suppression pulse was performed during the relaxation delay (RD). The number of transients was typically 64. The acquisition time was fixed to 3.1982555 s, and a quantity of four dummy scans was chosen.

The data were processed with Bruker TOPSPIN 3.5 software with a standard parameter setting. The phase and baseline corrections were performed manually over the entire spectral range. HPβCD quantification by ^{1}H NMR was performed as described by Dufour et al. [64]. The signals at 1.1 and 5.2 ppm were used for the quantification.

Extracellular analysis: A total volume of 1 mL of the extracellular medium was pipetted at the beginning (t_0 = 0 h) and at the end (t_1 = 2 h) of the incubation of A549 cells with HPβCD or the BUD:HPβCD complex in HBSS. The same operation was carried out in cell-free medium to subtract signals unrelated to the cellular uptake in the results. In total, 500 µL of collected culture media were supplemented with 100 µL of deuterated phosphate buffer (pH 7.4), 100 µL of a 35 mM solution of

maleic acid and 10 μL of TMSP. The solution was distributed into 5-mm tubes for NMR measurement. ^{1}H NMR spectra were acquired using a 1D NOESY sequence with presaturation. The NOESY presaturation experiment used an RD-90°-t1-90°-tm-90°-acquire sequence with a relaxation delay of 4 s, a mixing time (tm) of 10 ms and a fixed t1 delay of 4 μs. The water suppression pulse was performed during the relaxation delay (RD). The number of transients was 32, and a quantity of four dummy scans was chosen. The acquisition time was fixed to 3.2769001 s. The data were processed with Bruker TOPSPIN 3.5 software with a standard parameter setting. The phase and baseline corrections were performed manually over the entire spectral range. HPβCD quantification by ^{1}H NMR was performed as described by Dufour et al. [64] with the signal at 1.1 ppm.

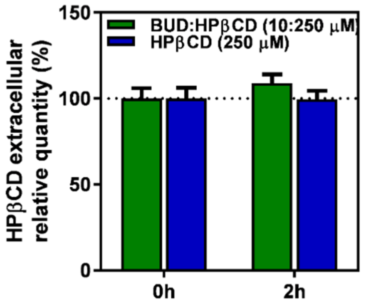

Figure S5. HPβCD relative quantity in A549 extracellular medium after 2 h of incubation with the BUD:HPβCD complex (green bars) and HPβCD (blue bars). HPβCD was quantified using proton nuclear magnetic resonance spectroscopy. Each bar represents the mean ± SEM of 3 independent measures. The results were normalized relative to HPβCD relative quantity at t_0 = 0 h (100 %). HPβCD relative quantity in cell-free medium was subtracted from HPβCD relative quantity in extracellular medium to exclude changes unrelated to cellular uptake.

Effect of NAC and Vit C on H_2O_2 + LPS-induced oxidant generation

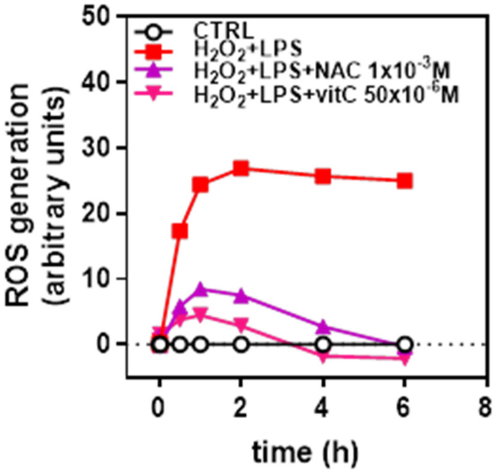

Figure S6. Oxidant generation kinetic in A549 cells after treatment with H_2O_2 + LPS (1 mM + 100 μg/mL) for 6 h and effect of *N*-acetyl-L-cysteine (NAC) and Vit C on H_2O_2 + LPS-induced oxidant generation. Oxidant generation was evaluated by measuring the fluorescence of dichlorofluorescein (DCF) resulting from the oxidation of DCFH2. The results were normalized relative to untreated cells (0) at each measure time. Each point represents the mean of triplicated measures (N = 1).

Akt phosphorylation induced by H_2O_2 + LPS, H_2O_2 and LPS in A549 cells after 2 h of incubation

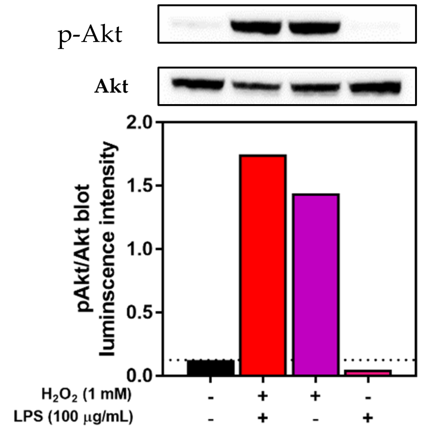

Figure S7. Akt phosphorylation induced by H_2O_2 + LPS, H_2O_2 and LPS in A549 cells after 2 h of incubation. Akt phosphorylation was quantified after a Western blot by measuring the proportion of phosphorylated-Akt (p-Akt) blot luminescence intensity/total Akt (Akt) blot luminescence intensity (N = 1).

Role of HDAC2 in the protection afforded by the BUD+ HPBCD on IL-8 release

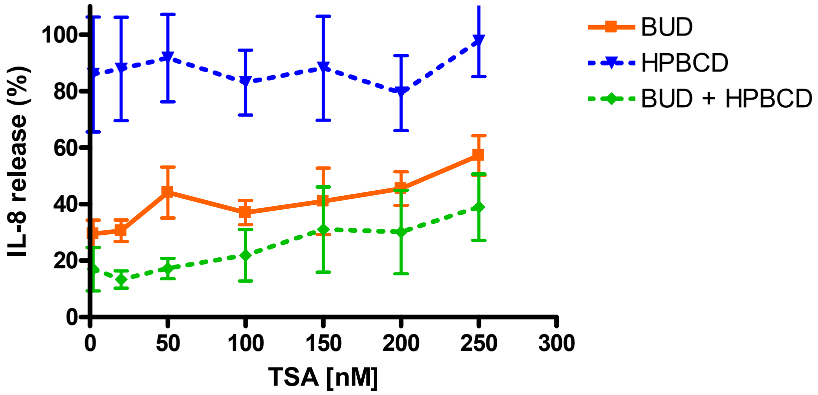

Figure S8. Percentage of IL-8 released after A 549 cells pretreatment for 30 min with trichostatin (TSA) and incubation for 2 h with TNF-α in presence of BUD + HPβCD complex, BUD or HPβCD. Results are expressed in percentage of IL-8 released induced by TNFβ-α. 100% corresponds to cells preincubated for 30 min with TSA and incubated for 2 h with TNF-α only. IL-8 release was measured in the extracellular medium by sandwich ELISA (N=2).

References

68. Pick, E.; Keisari, Y. A simple colorimetric method for the measurement of hydrogen peroxide produced by cells in culture. *J Immunol. Methods*. **1980**, *38*, 161–170.
69. Bahorun, T.; Gressier, B.; Trotin, F.; Brunet, C.; Dine, T.; Luyckx, M.; Vasseur, J.; Cazin, M.; Cazin, J.C.; Pinkas, M. Oxygen species scavenging activity of phenolic extracts from hawthorn fresh plant organs and pharmaceutical preparations. *Arzneimittelforschung*. **1996**, *46*, 1086–1089.
70. Brand-Williams, W.; Cuvelier, M.E.; Berset, C. Use of a free radical method to evaluate antioxidant activity. *LWT-Food Sci Technol*. **1995**, *28*, 25–30.
71. Matheus, N.; Hansen, S.; Rozet, E.; Peixoto, P.; Maquoi, E.; Lambert, V.; Noel, A.; Frederich, M.; Mottet, D.; de, T.P. An easy, convenient cell and tissue extraction protocol for nuclear magnetic resonance metabolomics. *Phytochem. Anal.* **2014**, *25*, 342–349.
